# Neuronal aldosterone elicits a distinct genomic response in pain signaling molecules contributing to inflammatory pain

**DOI:** 10.1186/s12974-020-01864-8

**Published:** 2020-06-12

**Authors:** Mohammed Shaqura, Li Li, Doaa M. Mohamed, Xiongjuan Li, Sascha Treskatsch, Constanze Buhrmann, Mehdi Shakibaei, Antje Beyer, Shaaban A. Mousa, Michael Schäfer

**Affiliations:** 1 Department of Anaesthesiology and Intensive Care Medicine, Charité – University Berlin, Corporate Member of Freie Universität Berlin, Humboldt-Universität zu Berlin, and Berlin Institute of Health, Campus Benjamin Franklin, Berlin, Germany; 2 Department of Zoology, Faculty of Science, Aswan University, Tingar, Egypt; 3 Department of Anesthesiology, Second Affiliated Hospital of Guangzhou Medical University, No. 250, Hai’zhu District, Guangzhou, 510260 China; 4 Department of Anatomy, Ludwig-Maximilians-University Munich, Munich, Germany; 5 Department of Anaesthesiology, Ludwig-Maximilians-University Munich, Munich, Germany

**Keywords:** Aldosterone synthase, Pain signaling molecules, Sensory neurons

## Abstract

**Background:**

Recently, mineralocorticoid receptors (MR) were identified in peripheral nociceptive neurons, and their acute antagonism was responsible for immediate and short-lasting (non-genomic) antinociceptive effects. The same neurons were shown to produce the endogenous ligand aldosterone by the enzyme aldosterone synthase.

**Methods:**

Here, we investigate whether endogenous aldosterone contributes to inflammation-induced hyperalgesia via the distinct genomic regulation of specific pain signaling molecules in an animal model of Freund’s complete adjuvant (FCA)-induced hindpaw inflammation.

**Results:**

Chronic intrathecal application of MR antagonist canrenoate-K (over 4 days) attenuated nociceptive behavior in rats with FCA hindpaw inflammation suggesting a tonic activation of neuronal MR by endogenous aldosterone. Consistently, double immunofluorescence confocal microscopy showed abundant co-localization of MR with several pain signaling molecules such as TRPV1, CGRP, Nav1.8, and trkA whose enhanced expression of mRNA and proteins during inflammation was downregulated following i.t. canrenoate-K. More importantly, inhibition of endogenous aldosterone production in peripheral sensory neurons by continuous intrathecal delivery of a specific aldosterone synthase inhibitor prevented the inflammation-induced enhanced transcriptional expression of TRPV1, CGRP, Nav1.8, and trkA and subsequently attenuated nociceptive behavior. Evidence for such a genomic effect of endogenous aldosterone was supported by the demonstration of an enhanced nuclear translocation of MR in peripheral sensory dorsal root ganglia (DRG) neurons.

**Conclusion:**

Taken together, chronic inhibition of local production of aldosterone by its processing enzyme aldosterone synthase within peripheral sensory neurons may contribute to long-lasting downregulation of specific pain signaling molecules and may, thus, persistently reduce inflammation-induced hyperalgesia.

## Introduction

Apart from their role of maintaining the homeostasis of the body through regulation of numerous physiologic processes [1, 2], there is increasing evidence for the presence and functional role of glucocorticoid (GR) and mineralocorticoid receptors (MR) within the central nervous system [3–5]. Both genomic and non-genomic effects of these steroid receptors were described contributing to an enhanced plasticity of the central nervous system [3–5].

More recent findings from our laboratory identified MR mainly on peripheral, CGRP-immunoreactive nociceptive neurons [6], and GR on peripheral and spinal nociceptive neurons [7], whereas they only scarcely colocalized with spinal glia cells or astrocytes. The fact that GR and MR are localized predominantly on peripheral and/or spinal nociceptive neurons suggests a pivotal functional role in the processing of painful stimuli. Indeed, application of GR agonists and MR antagonists attenuated nociceptive behavior [7, 8]. This effect occurred immediately and lasted only up to 60 min indicating a non-genomic effect.

Surprisingly, the mRNA and protein of enzyme aldosterone synthase, the enzyme that processes the last step of conversion to aldosterone, were detected in the same nociceptive neurons [9]. Immunohistochemical double-staining showed great overlap between aldosterone synthase, aldosterone, and MR in peripheral nociceptive neurons. Following a localized painful hindpaw inflammation, the expression of aldosterone synthase and the production of aldosterone were upregulated [9]. Intrathecal administration of the MR antagonist canrenoate-K resulted in an immediate and short-lasting (up to 30 min) reversal of mechanical hypersensitivity most likely through a non-genomic effect [9].

In contrast to this short-lasting effect, there is no systematic investigation on the putative genomic effects of MR on nociception, particularly on the transcriptional regulation of certain pain signaling molecules within peripheral nociceptive neurons which contribute to inflammation-induced hyperalgesia. Covenas et al. [10] provided the first evidence supporting the notion that MR may stimulate neuronal peptide synthesis. Moreover, aldosterone selectively increased Na + -K + -ATPase mRNA expression in rat hippocampus [11], and chronic intra-cerebroventricular infusion of aldosterone synthase inhibitor FAD286 or MR blocker eplerenone reduced epithelial sodium channel c subunit expression in supraoptic nucleus [12] and hypothalamus [13].

Thus, this study investigated in rats with Freund’s complete adjuvant-induced hind paw inflammation: (i) whether chronic i.t. application of the MR antagonist canrenoate-K attenuates nociceptive behavior, (ii) whether there is great overlap of MR with specific pain signaling molecules and whether their expression is affected by MR receptor blockade, (iii) whether the continuous inhibition of aldosterone’s production by an i.t. aldosterone synthase inhibitor will reverse the enhanced expression of pain signaling molecules and subsequently attenuate inflammation-induced hyperalgesia, and (iv) finally, whether the increased production of endogenous aldosterone during FCA hindpaw inflammation leads to enhanced nuclear translocation of MR supporting the occurrence of genomic effects.

## Material and methods

### Drugs

Freund’s complete adjuvant (FCA), a water-in-oil emulsion of killed mycobacteria (Calbiochem, San Diego, CA), isoflurane (Abbott, Wiesbaden, Germany), MR selective antagonist canrenoate-K, and aldosterone synthase inhibitor FAD286 were commercially obtained from Sigma-Aldrich (St. Louis, MO, USA) and were dissolved in 0.9% NaCl before application. Drugs were administered intrathecally either as single shot or via an intrathecal (i.t.) catheter as a continuous delivery using Alzet minipumps (Alzet Corporation, Cupertino, CA).

### Animals

Following approval by the local animal care committee and according to the European Directive (2010/63/EU) introducing new animal welfare and care guidelines, experiments were performed in male Wistar rats (180–250 g) (breeding facility Charité-Universitätsmedizin Berlin, Germany). Wistar rats received an intraplantar (i.pl.) injection of 0.15 ml FCA into the right hind paw under brief isoflurane (1.0–2.5 Vol%) anesthesia. This treatment consistently produces a local inflammation restricted to the inoculated paw characterized by an increase in paw volume, paw temperature, mechanical hypersensitivity, and infiltration of various types of immune cells as described previously [14]. All experiments were performed on the fourth day of FCA hindpaw inflammation.

### Experimental protocols

The first set of experiments examined the potential colocalization of MR with the pain signaling molecules TRPV1, CGRP, Nav1.8, and trkA in sensory L3-5 DRG neurons of naïve control rats. A second set of experiments assessed the impact of i.t. injection of 40 μg/20 μl of aldosterone (over four consecutive days) in naïve rats or 100 μg/20 μl of the MR antagonist canrenoate-K (over four consecutive days) in rats with FCA hindpaw inflammation on mechanical paw pressure thresholds (PPT). The i.t. doses chosen are too low to reach effective plasma concentrations [15, 16] and, thus, will not elicit any systemic effect [17]. The third set of experiments investigated the influence of 4 days FCA hindpaw inflammation on significant changes in the aldosterone, MR, TRPV1, CGRP, Nav1.8, and trkA expression in sensory L3-5 DRG neurons ipsilateral to the FCA hindpaw inflammation. The doses chosen for each drug were based on generated dose-response curves in our previous studies [6–8]. A final set of experiments investigated the effects of continuous i.t. delivery of the aldosterone synthase inhibitor FAD286 on mechanical PPT and neuronal TRPV1, CGRP, Nav 1.8, and trkA expression after 4 days FCA hindpaw inflammation. For i.t. FAD286 application, Alzet osmotic minipumps (2000 μl, rate 5 μl/h) were filled with 0.9% NaCl with or without 0.3ug/1 μ1 FAD286 and connected to the i.t. catheter to administer FAD286 or vehicle continuously at 5 μl/h; an i.t. dose of 1.5 μg/5 μl/h will not lead to effective plasma concentrations and, thus, will not elicit any systemic effect [18].

For i.t. drug administration, animals were anesthetized with isoflurane in oxygen via nose cone, and a longitudinal skin incision was made in the lumbar region directly above the spinous processes of the L3–L5 vertebrae as described previously [19]. Briefly, the i.t. needle was inserted at a 30° angle between the L4 and L5 vertebra into the i.t. space and either a single injection or the placement of an i.t. catheter (PE 10 tubing attached to PE 60 tubing for attachment to an osmotic pump; Portex Ltd, Hythe, Kent, United Kingdom) was set up. The sign of dura penetration was observed by involuntary movements of the tail or hind limb and verified the i.t. space for drug delivery.

### Mechanical hyperalgesia testing

The mechanical hyperalgesia following FCA hindpaw inflammation was assessed by a mechanical pressure apparatus (Ugo-Basile SRL, Monvalle, Italy) with increasing force (measured in grams) applied to the plantar hind paw until a withdrawal reflex was precipitated as described previously [8]. Mechanical paw pressure thresholds (PPT) which triggered a withdrawal response were determined in all groups on the fourth day of FCA hindpaw inflammation. Drug application baseline values were obtained in both inflamed and contralateral non-inflamed hind paws. Then, the PPT were reassessed 2 h following the last i.t. drug administration to determine drug-related behaviors. PPT measurements were performed 3 times consecutively. The final PPT were calculated as the mean obtained from 6–7 animals before and after i.t. drug administration. In all behavioral experiments, drugs were prepared by a different person (M.Sh.), and the examiner (X.L.) was unaware of the treatment that each animal received by chance.

### TRPV1, CGRP, Nav1.8, and trkA mRNA detection by quantitative RT-PCR

Total RNA was extracted from L3-5 dorsal root ganglia of Wistar rats (*n* = 5 per experimental group) using RNeasy Kit (Qiagen, Hilden, Germany) as previously described [8, 20]. The following specific primers were generated and used: for TRPV1, forward primer: AGTGAGACCCCTAACCGTCA, reverse primer: CGGAAATAGTCCCCAACGGT (Ensembl, Accession Nr: NM_031982.1); for CGRP, forward primer: CCTTTCCTGGTTGTCAGCATCTT, reverse primer: CAGTAGGCG AGCTTCTTCTTCAC (Ensembl, Accession NM_001033956.1); for Nav.1.8, forward primer: CACGGATGACAACAGGTCAC, reverse primer; GATCCCGTCAGGAAATGAGA (Ensembl, Accession Nr: NM_017247.1); for trkA, forward primer: CCATCCCTGTCT CCTTCTCGC, reverse primer: CCCAAAAGGTGTTTCGTCCTTC (Ensembl, Accession Nr: NM_021589.1). Quantitative real-time PCR (RT-PCR) was performed with a SYBR® Green kit following the manufacturer’s instructions (Applied Biosystems, Carlsbad, CA). Amplification was carried out for 40 cycles, each consisting of 15 s at 95 °C. A temperature just below the specific melting temperature (Tm) was employed for detection of fluorescence specific products (TRPV1: Tm 76 °C, 18S: Tm 83 °C). TRPV1, CGRP, Nav1.8, and trkA mRNA were quantified using triplicates of samples using the delta-delta CT method [21, 22].

### Aldosterone content measurements in DRG

In deep isoflurane anesthesia rats (*n* = 7–8 per group) were sacrificed, L3-5 DRG were quickly removed in DMEM–Dulbecco’s Modified Eagle Medium (Thermo Fisher Scientific GmbH Berlin, Germany). DRG were prepared as described previously [8, 23]. Briefly, 1 mg/ml collagenase IV was added to the DRG and incubated for 30 min at 37 °C. To stop the collagenase activity, 0.05% trypsin was added and again incubated for 10 min at 37 °C. Finally, the sample was centrifuged at 500×*g* for 5 min at room temperature and the pellet resuspended in 1 ml RPMI 1640 (GibCO, Thermo Fischer, Dreieich, Germany). After mechanical cell lysis and centrifugation at 500×*g* for 5 min, the aldosterone content in DRG was determined by a commercial kit from R&D Systems (Minneapolis, MN, USA, Cat. # KGE016). For this, we followed the protocol according to the manufacture’s manual and our previous study [8]. Briefly, all reagents and samples were brought to room temperature before use. Determinations of aldosterone in the different samples and standards were done in triplicate as recommended by the manufacture. The optical density of each well was determined within 30 min using a microplate reader (Infinite M200, Tecan, Männedorf, Switzerland) set to 450 nm. Then, the average of the triplicate readings for each standard, control and sample were determined and subtracted by the average NSB optical density (O.D.). A standard curve was created using the computer software Magellan Vers. 7.2 (Tecan, Männedorf, Switzerland) capable of generating a four-parameter logistic (4-PL) curve-fit. The results were calculated by wet weight.

### Immunohistochemistry

After transcardial perfusion of rats L3-L5 DRG were removed and further processed as described previously [6, 8], slide mounted tissue sections (8 μm) were obtained by using a kryostat (Microm,Thermo Fischer, Dreieich, Germany). Tissue sections were then incubated overnight with the following primary antibodies (see also supplemental table 1): mouse antibody against MR (private gift from Prof. Elise Gomez-Sanchez, Jackson, USA) was examined alone or in combination with a polyclonal rabbit anti-Nav1.8 (Sigma-Aldrich, USA), guinea pig anti-CGRP, goat anti-trkA (R&D Systems, USA) or goat anti-TRPV1 (Santa Cruz Biotechnology, California): in addition, polyclonal rabbit anti-aldosterone (Novus Biologicals, CO, USA) alone or in combination with the polyclonal guinea pig anti-CGRP or monoclonal mouse anti-MR. The species, sources, dilutions, and immunogens of the primary antibodies used in this study are summarized in the supplemental table 1. Finally, the tissues were washed in PBS, mounted on vectashield (Vector Laboratories), and imaged with a confocal laser scanning microscope, LSM510 as described previously [8]. To demonstrate specificity of staining, the following controls were included as described in our previous studies [7, 8, 20]: omission of either the primary antisera or the secondary antibodies.

The quantification of DRG staining has been described previously [7, 8, 20]. Quantification of immunofluorescence of MR, TRPV1, CGRP, Nav1.8, and trkA in DRG tissue sections was performed by using the Zeiss Zen 2009 software Carl Zeiss Micro-Imaging GmbH (Göttingen, Germany). For counting of the total number of neurons, only those immunostained neurons containing a distinct nucleus were counted. In a similar way, the number of aldosterone-ir DRG cells/320μm^2^, MR-ir DRG cells/320μm^2^, TRPV1-ir DRG cells/320μm^2^, CGRP-ir DRG cells/320μm^2^, Nav1.8-ir DRG cells/320μm^2^, and trkA-ir DRG cells/320μm^2^ cells was counted in each DRG section and represented as percentages. Data were obtained from 4–6 rats per group using × 40 objective lens.

### Preparation and immunoblotting of cytosol and nuclear extract

The isolated DRG neurons from control or FCA treated animals were washed three times in ice cold HANKs solution (10,000 rpm/3 min) to remove any remaining medium or buffer from the previous step of cell isolation. For extraction of cytoplasma, the supernatent was carefully removed and the cell pellet resuspended in cytoplasmic extraction buffer (10 mM HEPES, 10 mM KCl, 0.1 mM EDTA, 0.1 mM EGTA) containing 0.1 mM DTT, 0.1 mM PMSF, 2 μg/ml leupetin, 2 μg/ml aprotinin, 0.5 mg/ml benzamidin, and incubated on ice for 30 min. Then, 10% NP-40 was added, each sample vortexed for 30 s, centrifuged (10 000 rpm/3 min), and the supernatant (cytosol extract) transferred to a new tube and stored at − 80 °C. For further preparation of the nuclear extract, the remaining pellet (containing the nuclei) was re-suspended in nuclear extraction buffer (20 mM HEPES, 400 mM NaCl, 1 mM EDTA, 1 mM EGTA) containing 1 mM DTT, 0.5 mM PMSF, 2 μg/ml leupetin, 2 μg/ml aprotinin, 0.5 mg/ml benzamidin, and incubated for 30 min on ice. Finally, samples were centrifuged (10,000 rpm/3 min), and supernatant containing nuclear extract was transferred to a new tube and stored at − 80 °C. The total protein content in samples was measured using the bicinchinonic acid system (Uptima, France), the samples were reduced with 2-mercaptoethanol, and the protein contents were adjusted. The samples were separated by SDS-PAGE electrophoresis (7% gels), transferred to a nitrocellulose membrane, washed for 2 h with skimmed milk buffer for unspecific blocking, and incubated with primary antibodies for mineralocorticoid receptor (rMR), Poly (ADP-ribose) polymerase (PARP), or β-actin overnight at 4 °C. Finally, the membranes were washed three times with skimmed milk buffer, incubated with secondary antibodies for 2 h, and antibody-antigen complexes visualized by nitro-blue tetrazolium and 5-bromo-4-chloro-3-indoylphosphate (p-toluidine salt; Pierce, Rockford, USA). Experiments were done in 6 animals per group in duplicate.

The Western blot band specific for rMR (107 kDa) or PARP (110 kDa) was quantified by the Java Image processing and analysis software (Image J, open-source image software downloaded from the web^1^ [6, 7]. The area and density of pixels within the threshold values representing immunoreactivity were measured, and the integrated density (the product of the area and mean of grey values) was calculated. Integrated immunodensities of controls and treated groups were compared and statistically analysed [19].

### Statistical analysis

All tests were performed using the Sigma Stat 2.03 software (SPSS Inc., Germany). Quantitative RT-PCR, Western blot, and immunofluorescence data were analyzed as two group comparisons (FCA treated rats versus controls) by a two-tailed independent Student *t* test in case of normally distributed data. Three group comparisons were done for quantitative RT-PCR and immunohistochemistry experiments by one-way ANOVA followed by a post-hoc Dunnett’s test. Paw pressure thresholds (PPT) were determined before and after drug injections within the same group of animals, expressed as means ± SD, and statistically analyzed by a repeated measurement-ANOVA, followed by post-hoc Dunnett’s test. For all statistical tests, significance was assumed at *P* < 0.05.

## Results

### Chronic antagonism of endogenous aldosterone or administration of exogenous aldosterone reveals aldosterone’s contribution to an enhanced mechanical hypersensitivity

**Fig. 1:**
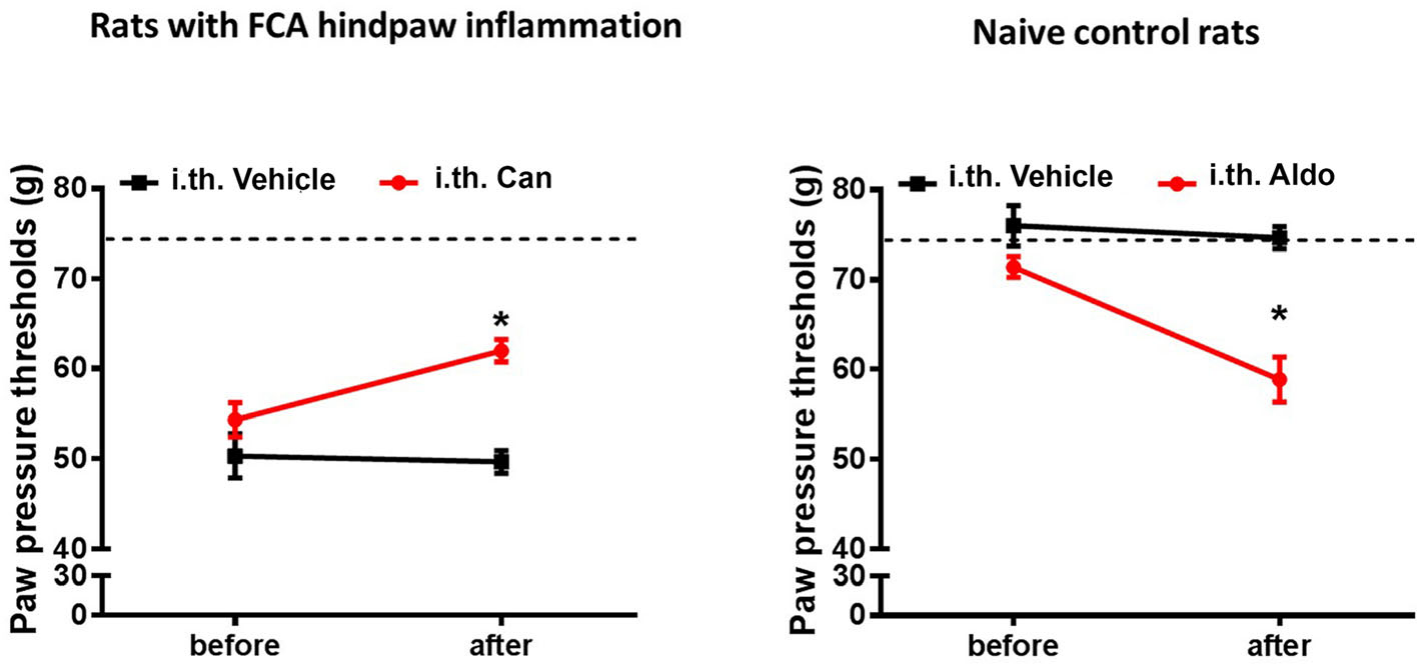
Role of mineralocorticoid receptor (MR) specific antagonist canrenoate-K (Can) in the modulation of Freund’s complete adjuvant (FCA) inflammation-induced nociceptive behavior. **a**, **b** Four days FCA inflammation of the right hindpaw resulted in significantly reduced mechanical PPT thresholds compared to baseline values (dotted line) (*P* < 0.05, repeated measurement-ANOVA, followed by post-hoc Dunnett’s test; *n* = 6). Chronic i.t. canrenoate-K reversed this mechanical hypersensitivity (*P* < 0.05, repeated measurement-ANOVA, followed by post-hoc Dunnett’s test; *n* = 6). Data are expressed as means ± SD. **b** In contrast, i.t. administration of repetitive doses of the exogenous aldosterone resulted in significant PPT reductions compared to baseline (0 min) indicating a reduced mechanical nociceptive thresholds (hyperalgesia) ( *P* < 0.05, repeated measurement-ANOVA, followed by post-hoc Dunnett’s test; *n* = 6). Data are expressed as means ± SD

Four days of FCA-induced inflammation of the right hindpaw resulted in diminished mechanical PPT thresholds in inflamed compared to non-inflamed hindpaws (dashed line, Fig. 1) (*P* < 0.05, repeated measurement-ANOVA, followed by post-hoc Dunnett’s test, Fig. 1a). This enhanced mechanical hypersensitivity in inflamed hindpaws was significantly attenuated following daily i.t. administrations of the MR selective antagonist canrenoate-K (*P* < 0.05, repeated measurement-ANOVA, followed by post-hoc Dunnett’s test; Fig. 1a). Canrenoate-K had no effect on the contralateral non-inflamed hindpaw (data not shown), similar to previous reports in naive rats [8]. Conversely in naïve control rats, repeated i.t. administration of the MR selective agonist aldosterone over 4 days resulted in a significant decrease of mechanical PPT thresholds and, thus, enhanced sensitivity to mechanical stimuli (*P* < 0.05, repeated measurement-ANOVA, followed by post-hoc Dunnett’s test; Fig. 1b), while vehicle administration did not significantly alter mechanical PPT (*P* < 0.05, repeated measurement-ANOVA, followed by post-hoc Dunnett’s test; Fig. 1b).

### Chronic intrathecal MR antagonism inhibits the enhanced transcription of pain signaling molecules colocalizing with MR during inflammatory pain

**Fig. 2:**
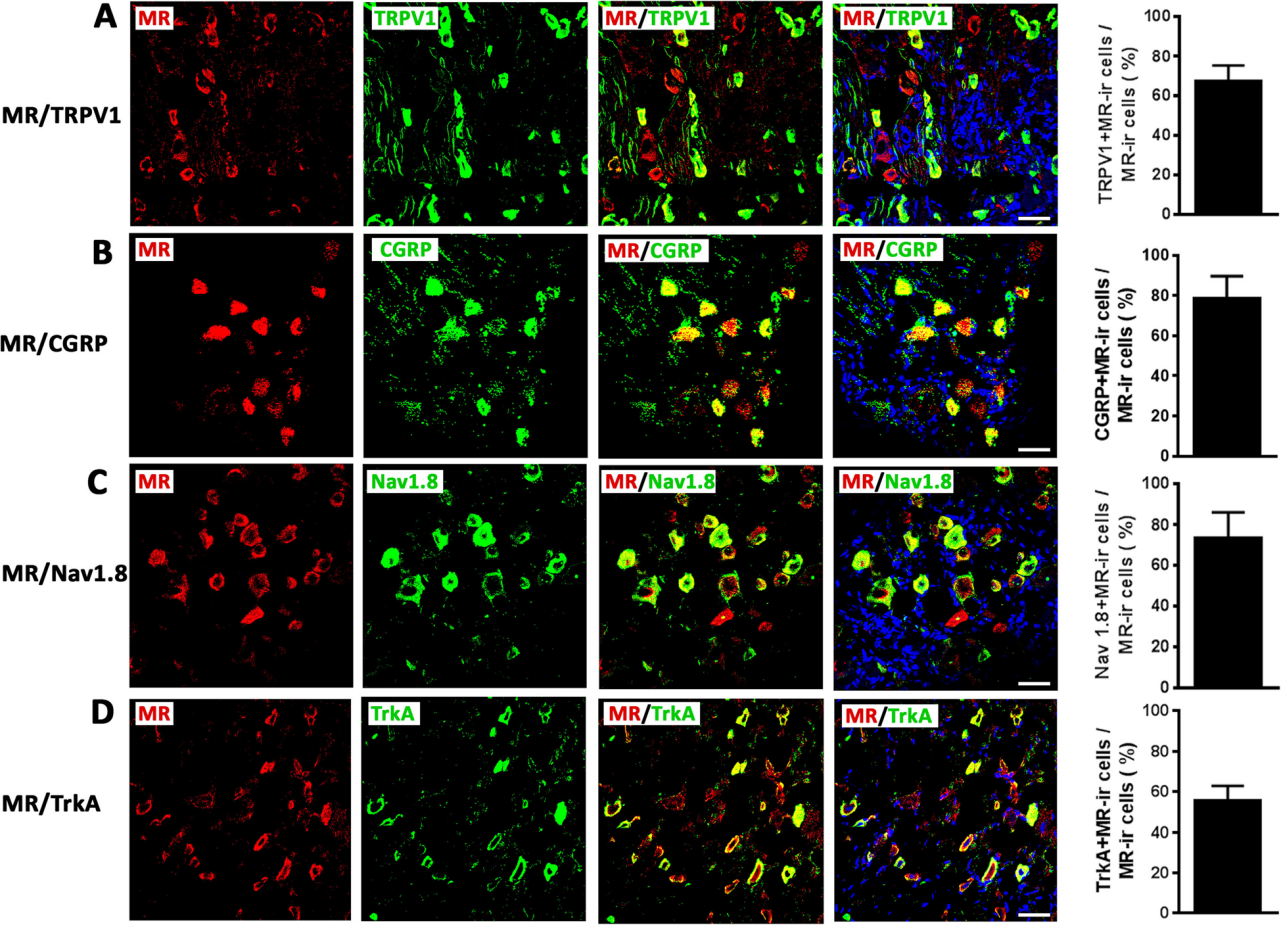
Detection of mineralocorticoid receptor (MR) with pain signaling molecules TRPV1, CGRP, Nav1.8, and trkA in dorsal root ganglia (DRG) of control rats. **a**–**d** Double immunofluorescence confocal microscopy of MR (*red fluorescence)* with TRPV1, CGRP, Nav1.8, and trkA (*green fluorescence)* in DRG sections (Bar = 40 μm). **a**–**d** The majority of MR-IR DRG sensory neuron express TRPV1, CGRP, Nav1.8, and trkA in control animals (Bar = 40 μm). Data are expressed as means ± SD; *n* = 7–12

**Fig. 3:**
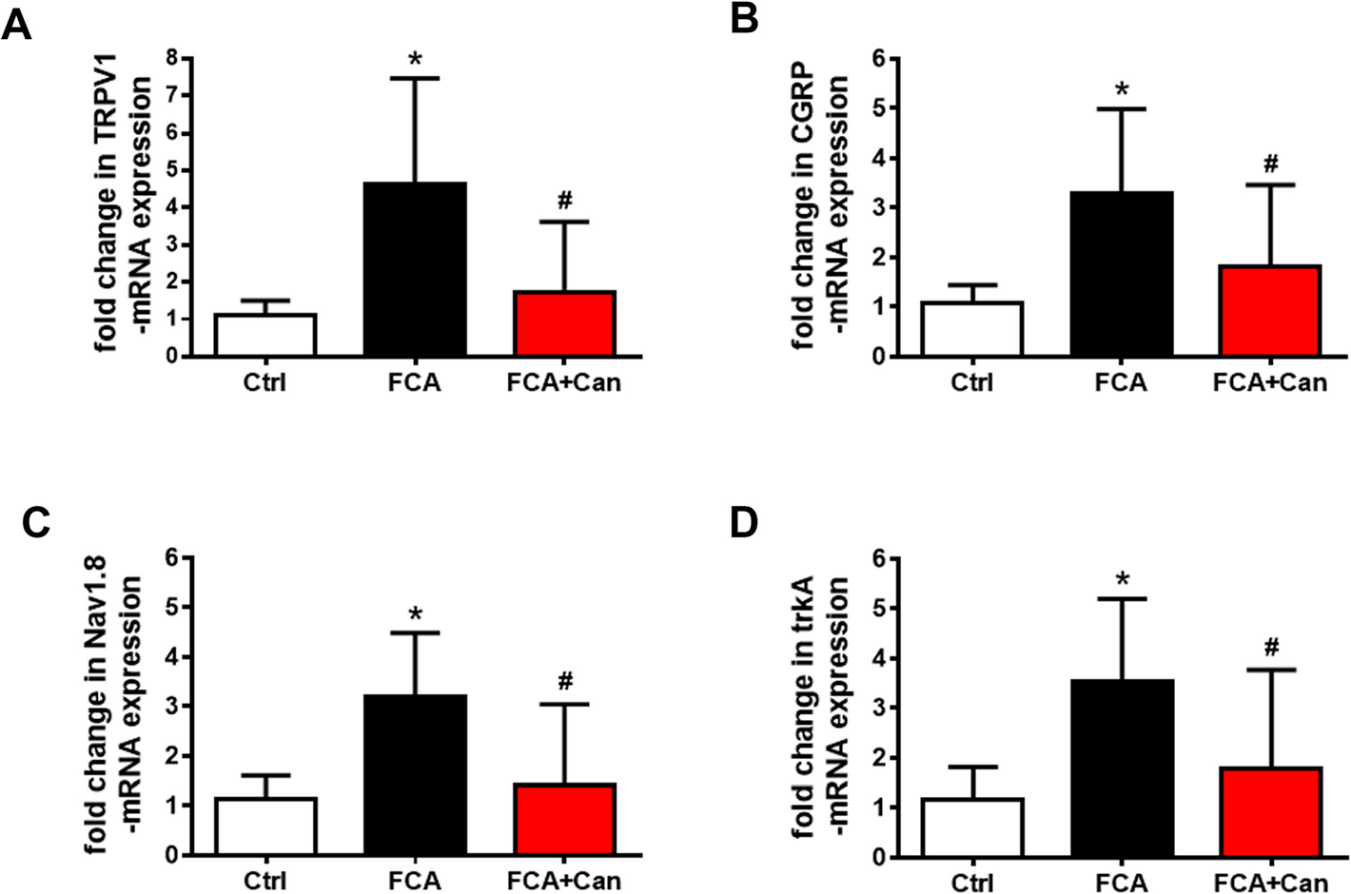
MR selective antagonist canrenoate-K reversed the enhanced expression of pain signaling molecules TRPV1 (**a**), CGRP (**b**), Nav1.8 (**c**), and trkA (**d**) mRNA in nociceptive DRG neurons with inflammatory pain. **a**–**d** Chronic i.t. canrenoate-K administration to rats with Freund’s complete adjuvant (FCA)-induced inflammation reversed the enhanced expression of pain signaling molecules TRPV1 (**a**), CGRP (**b**), Nav1.8 (**c**), and trkA (**d**) mRNA. (*P* < 0.05, one-way ANOVA, followed by post-hoc Dunnett’s test, *n* = 10). Data are expressed as means ± SD

**Fig. 4:**
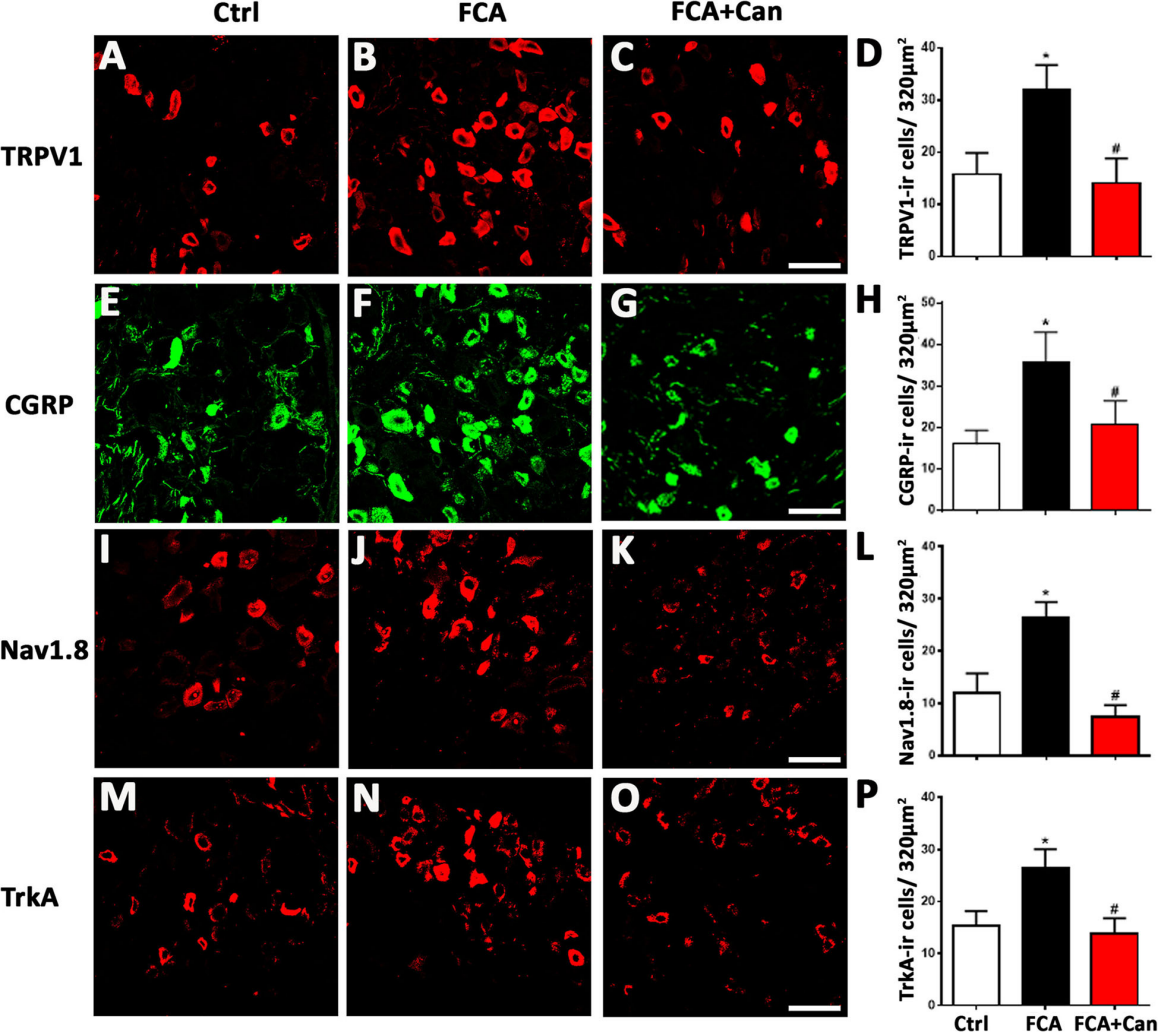
MR selective antagonist canrenoate-K reversed the enhanced expression of pain signaling molecules TRPV1 (**a**), CGRP (**b**), Nav1.8 (**c**), and trkA (**d**) immunoreactivity in nociceptive DRG neurons with inflammatory pain. **a**–**d** Chronic i.t. canrenoate-K administration to rats with Freund’s complete adjuvant (FCA)-induced inflammation reversed the increased number of pain signaling molecules TRPV1 (**a**), CGRP (**b**), Nav1.8 (**c**), and trkA (**d**) IR DRG neurons. (*P* < 0.05, one-way ANOVA, followed by post-hoc Dunnett’s test, *n* = 9–17) (Bar = 40 μm). Data are expressed as means ± SD

Our double immunofluorescence confocal microscopy showed abundant colocalization of MR with several pain signaling molecules such as TRPV1, CGRP, Nav1.8, and trkA (TRPV1 68%; CGRP 79%; Nav1.8 74 %; trkA 56%) (Fig. 2). To investigate potential genomic effects of MR on the expression of these pain signaling molecules, we repeatedly treated rats with FCA hindpaw inflammation over 4 days with i.t. administration of the MR antagonist canrenoate-K (Fig. 3a–d). Indeed, the significantly enhanced mRNA expression of pain signaling molecules TRPV1, CGRP, Nav1.8, and trkA in sensory DRG ipsilateral to the FCA hindpaw inflammation was significantly attenuated by chronic i.t. canrenoate-K delivery (*P* < 0.05, one-way ANOVA, followed by Dunnett’s test; Fig. 3a–d). Consistent with these findings, the significantly increased numbers of TRPV1-, CGRP-, Nav1.8-, and trkA-immunoreactive DRG neurons ipsilateral to the FCA hindpaw inflammation were significantly reduced by chronic i.t. canrenoate-K administration (*P* < 0.05, one-way ANOVA, followed by Dunnett’s test; Fig. 4a–p), although the total number of DRG cells was not significantly altered (Ctrl 45 ± 8.5; FCA 45 ± 8.8; FCA + Can 42 ± 12, *P* = 0.43 one-way ANOVA).

### Enhanced MR translocation to the nucleus of DRG neurons during inflammatory pain

**Fig. 5:**
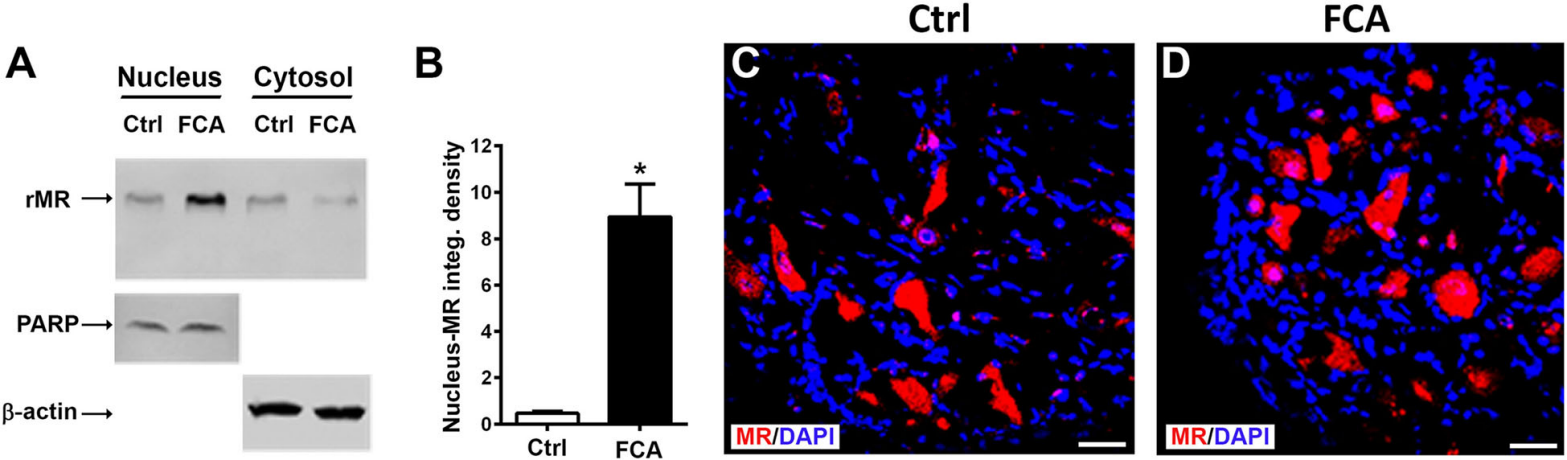
Western blot of whole protein extracts of dorsal root ganglia (DRG) nuclei and cytosol fractions of rats with hindpaw inflammation versus control (Ctrl) with anti-mineralocorticoid receptor (MR), Poly(ADP-ribose)polymerase (PARP), or ß-actin antibody. **a**, **b** Western blots of the nuclear and cytosol fractions were verified by the detection of the nuclear component Poly(ADP-ribose)polymerase (PARP) (110 kDa) and the cytosol component ß-actin (43 kDa), respectively. In both the nuclear and cytosol fractions MR (107 kDa), specific protein was detected under inflamed and control conditions. However, nuclear fractions from DRG innervating inflamed hindpaws showed a significant 8-fold increase in the density of the MR protein compared to control DRG (*P* < 0.05, two-tailed independent Student’s *t* test; *n* = 6). **c**, **d** Confocal microscopy showing increasing overlap of MR-immunorecativity (red fluorescence) with nuclear DAPI (blue fluorescence) staining of DRG of FCA-treated rats versus controls

Following several steps of DRG cell extract separation into a nuclear and cytosolic fraction subsequent western blot analysis identified a MR specific protein band at 107 kDa in both fractions (Fig. 5a, b). The detection of PARP (110 kDa), which is a nuclear specific enzyme for DNA repair, validated the nuclear fraction, whereas the detection of ß-actin verified the cytosolic fraction (Fig. 5a, b). In the nuclear but not cytosolic fraction, DRG innervating the inflamed hindpaws of rats showed a significant increase in the density of the MR protein band compared to those DRG innervating non-inflamed hindpaws (*P* < 0.05, Student’s *t* test; Fig. 5a, b). This was confirmed by immunohistochemistry of the respective DRG showing an increasing overlap of MR-immunoreactivity with the nuclear DAPI staining indicating enhanced nuclear translocation following 4 days of FCA-induced hindpaw inflammmation.

### Chronic intrathecal aldosterone synthase inhibition prevents the enhanced expression of pain signaling molecules and the elevated mechanical sensitivity during inflammatory pain

**Fig. 6:**
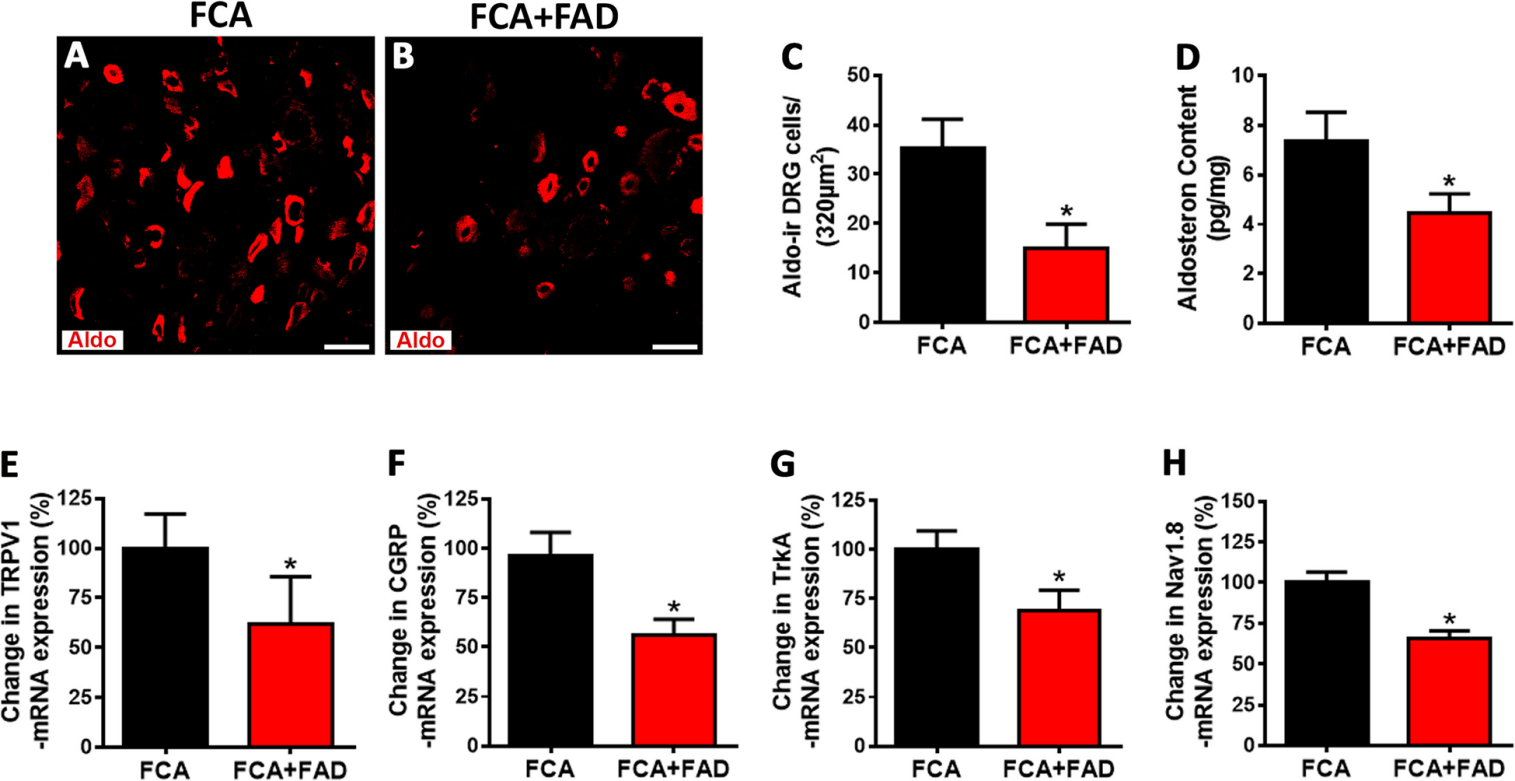
Aldosterone synthase inhibitor FAD286 reversed the elevated aldosterone content as well as enhanced expression of pain signaling molecules TRPV1, CGRP, Nav1.8, and trkA mRNA in nociceptive dorsal root ganglia (DRG) neurons with inflammatory pain. **a**, **b** Determination of aldosterone-immunoreactive (IR) DRG neurons of FCA rats treated with i.t. aldosterone synthase inhibitor FAD286. Note, the number of aldosterone-IR DRG neurons of FCA rats was increased by approximately 2.2-fold compared to controls which was reversed following continuous i.t. aldosterone synthase inhibitor FAD286 treatment over 5 days (**b**) (*P* > 0.05, two-tailed independent Student’s *t* test; *n* = 7–11). **c** Determination of aldosterone content in DRG of FCA rats treated with i.t. aldosterone synthase inhibitor FAD286 by a fluorometric enzyme-linked immunoassay. Note, aldosterone content in DRG of FCA rats was increased by approximately 2.6-fold compared to control which was reversed following i.t. adosterone synthase inhibitor FAD286 treatment (*P* < 0.05, two-tailed independent Student’s *t* test; *n* = 7,8). **e**–**h** Long-lasting i.t. aldosterone synthase inhibitor FAD286 to rats with Freund’s complete adjuvant (FCA)-induced inflammation reversed the enhanced expression of pain signaling molecules TRPV1 (**e**), CGRP (**f**), Nav1.8 (**g**), and trkA (**h**) mRNA. (*P* < 0.05, two-tailed independent Student’s *t* test; *n* = 5). Data are expressed as means ± SD

**Fig. 7:**
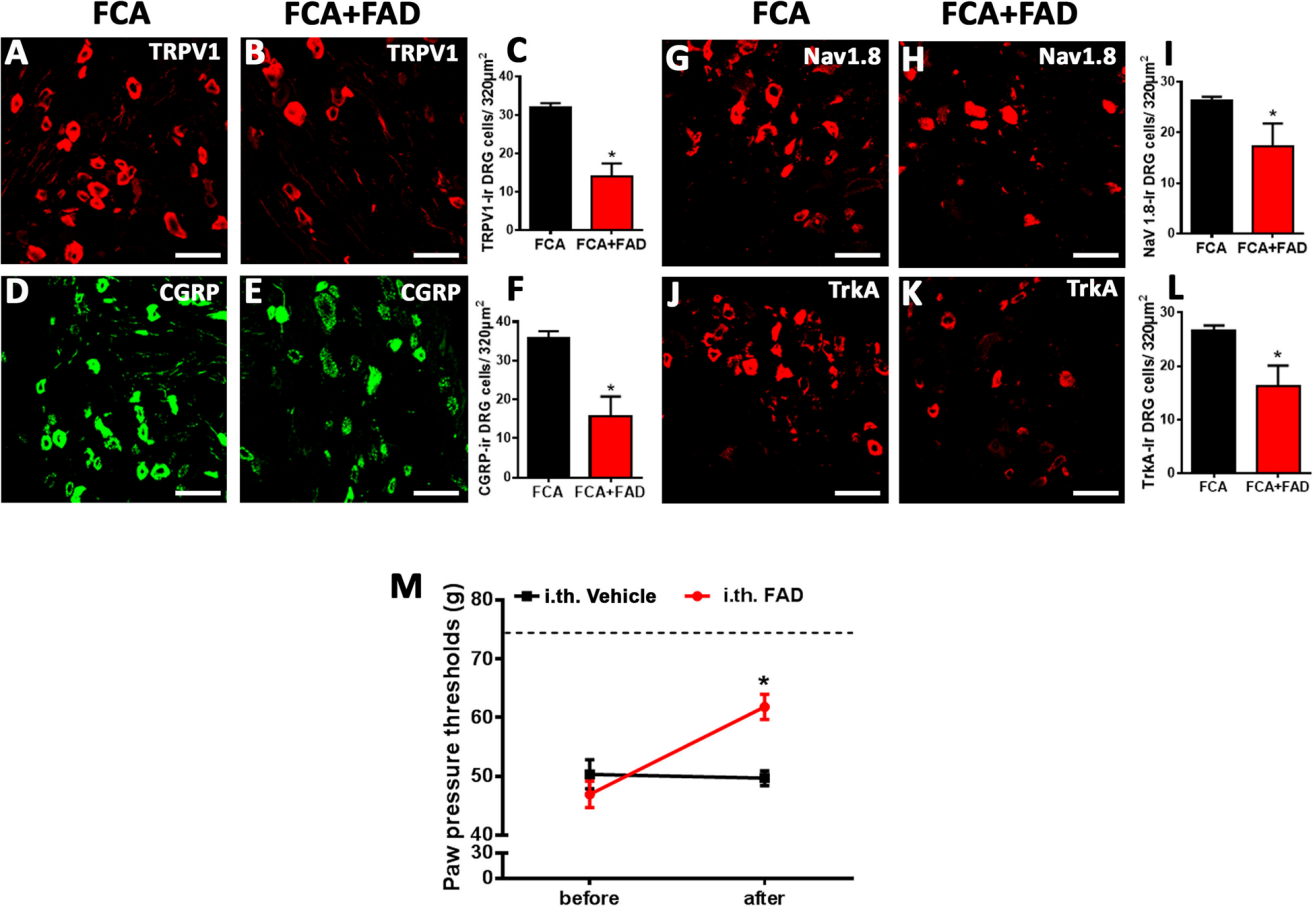
Aldosterone synthase inhibitor FAD286 reversed the enhanced expression of pain signaling molecules TRPV1, CGRP, Nav1.8, and trkA immunoreactivity in nociceptive dorsal root ganglia (DRG) neurons as well as mechanical hyperalgesia during inflammatory pain. **a**–**l** Continuous i.t. aldosterone synthase inhibitor FAD286 infusion to rats with Freund’s complete adjuvant (FCA)-induced inflammation reversed the increased number of pain signaling molecules TRPV1- (**a**–**c**), CGRP- (**d**–**f**), Nav1.8- (**g**–**i**), or trkA- (**j**–**l**) IR DRG neurons. (*P* < 0.05, two-tailed independent Student’s *t* test; *n* = 9–17) (Bar = 40 μm). Data are expressed as means ± SD. **m** Continuous i.t. aldosterone synthase inhibitor FAD286 infusion resulted in significant PPT elevations compared to baseline (0 min) indicating a reversal of FCA inflammation-induced mechanical hyperalgesia (*P* < 0.05, repeated measurement-ANOVA, followed by post-hoc Dunnett’s test; *n* = 6–10)

Since aldosterone synthesized in peripheral sensory DRG neurons seemed to contribute to inflammatory pain in rats with FCA hindpaw inflammation, we examined whether continuous i.t. infusion of aldosterone synthase inhibitor FAD286 diminished aldosterone synthesis in DRG of inflamed rats to that of controls. Indeed, i.t. infusion of FAD286 but not vehicle in FCA treated rats significantly reduced the number of aldosterone-IR DRG neurons determined by immunofluorescence confocal analysis (*P* < 0.05, Student’s *t* test; Fig. 6a–c), although the total number of DRG cells was not significantly altered (Ctrl 45 ± 8.5; FCA 45 ± 8.8; FCA + FAD 40 ± 6.7, *P* = 0.433 one-way ANOVA). Moreover, aldosterone specific ELISA experiments revealed that the increase in aldosterone DRG content of FCA animals was also significantly diminished following i.t. aldosterone synthase inhibitor FAD286 (*P* < 0.05, Student’s *t* test; Fig. 6d). To verify whether the reduced aldosterone content in DRG would affect the enhanced expression of the pain signaling molecules TRPV1, CGRP, Nav1.8, and trkA following FCA hindpaw inflammation, we examined DRG neuron mRNA as well as the number of DRG neurons containing these molecules following chronic i.t. administration of aldosterone synthase inhibitor FAD286. This treatment significantly prevented the inflammation-induced increase in mRNA (*P* < 0.05, Student’s *t* test; Fig. 6e–h) and in the number of TRPV1-, CGRP-, Nav1.8-, and trkA-IR nociceptive DRG neurons (*P* < 0.05, Student’s *t* test; Fig. 7a–l). Concomitant with this prevention, the FCA-induced enhanced mechanical hypersensitivity was significantly reduced following i.t. FAD286 (*P* < 0.05, Student’s *t* test; Fig. 7m).

## Discussion

For the first time, we have systematically investigated the potential long-lasting genomic effects of endogenous MR agonist aldosterone on the transcriptional regulation of several pain signaling molecules in peripheral sensory DRG neurons during inflammatory pain. Previous studies have demonstrated MR and its endogenous ligand aldosterone in these neurons [8, 9]. Here, chronic i.t. antagonism of endogenous aldosterone during hindpaw inflammation or, conversely, chronic application of exogenous aldosterone in animals without hindpaw inflammation attenuated or evoked mechanical hypersensitivity, respectively. Since the i.t. doses chosen are too low to elicit systemic effects, the findings suggest a persistent endogenous tonic activation of neuronal MR during local inflammation. Interestingly, double immunofluorescence confocal microscopy showed abundant colocalization of MR with several pain signaling molecules such as TRPV1, CGRP, Nav1.8, and trkA. Moreover, continuous (over 4 days) inhibition of endogenous aldosterone production in peripheral sensory neurons by i.t. application of the specific aldosterone synthase inhibitor FAD286 prevented the enhanced transcriptional expression of pain signaling molecules TRPV1, CGRP, Nav1.8, and trkA and subsequently attenuated the nociceptive behavior during hindpaw inflammation. This genomic effect of endogenous aldosterone during inflammatory pain was supported by the demonstration of an enhanced translocation of MR from the cytosol to the nucleus in peripheral sensory DRG neurons.

Previously, we have demonstrated that the final conversion of 18-hydroxycorticosterone into aldosterone by aldosterone synthase also occurs in peripheral sensory DRG neurons [9] confirming preceding reports on the expression of aldosterone synthase outside the adrenal gland, e.g., in brain, heart, aortic endothelial cells, and in vascular smooth muscle [24–26]. Moreover, we have shown that a single systemic or intrathecal administration of the MR antagonist canrenoate-K resulted in an immediate and short-lasting (up to 30 min) reversal of mechanical hypersensitivity most likely through a non-genomic effect [9]. In contrast to this short-lasting effect, we investigated in this study whether chronic antagonism of aldosterone’s action or continuous inhibition of aldosterone synthesis (over 4 days) resulted in alterations of the genomic expression of specific pain signaling molecules such as TRPV1, CGRP, Nav1.8, and trkA and whether this resulted in changes of nociceptive behavior following FCA-induced hindpaw inflammation.

First, we demonstrated that chronic i.t. administration of the MR selective antagonist canrenoate-K attenuated mechanical hypersensitivity in inflamed hindpaws suggesting a tonic activation of neuronal MR by endogenous aldosterone. Conversely, chronic i.t. administration of exogenous aldosterone in naïve rats resulted in mechanical hypersensitivity. Previous studies have demonstrated that the MR-immunoreactivity within the dorsal horn of the spinal cord mainly derives from peripherally incoming CGRP-ir nociceptive neurons in Rexed laminae I and II, and only few scattered neuronal cells were identified in Rexed laminae III and IV [6]. Although the corticosterone inactivating enzyme 11ßHSD2 colocalized with sensory neuron MR allows MR activation predominantly by aldosterone [8], the co-activation by corticosterone cannot be fully excluded. Our findings are in agreement with older studies which have shown a long-lasting analgesic effect of MR antagonists [27, 28].

Then, we found that several potential pain signaling molecules such as TRPV1, CGRP, Nav1.8, and trkA showed a 60–75% overlap with MR-immunoreactive DRG neurons, which is consistent with previous studies on the identification of certain subpopulations of MR expressing peripheral sensory neurons [6, 7]. In general, literature on a potential colocalization of neuronal MR with pain signaling molecules is scarce. Derbenev et al. for example reported about a putative colocalization of GR with TRPV1, since whole-cell patch-clamp recordings in the dorsal nucleus of the vagal nerve showed GR-mediated activation of TRPV1 receptors on afferent terminals [29]. In line with older reports [10, 30, 31] about a coexistence of GR with the spinal neuropeptides substance P and CGRP, our previous studies have demonstrated that MR and GR colocalize with CGRP in DRG neurons [6, 7].

Furthermore, our data show that the expression of these pain signaling molecules TRPV1, CGRP, Nav1.8, and trkA is upregulated in DRG neurons ipsilateral to the local hindpaw inflammation consistent with numerous older studies [32–35]. Interestingly, chronic i.t. administration of the MR antagonist canrenoate-K in rats with FCA-induced hindpaw inflammation caused a downregulation of the mRNA and immunoreactivity of these pain signaling molecules in DG neurons suggesting that an intrinsic tonic activation of sensory neuron MR by endogenous aldosterone may contribute to this upregulation. Importantly, this downregulation in various pain signaling molecules was not caused by a decrease in the total number of DRG cells according to our results.

What is the most likely mechanism? It is widely accepted that aldosterone functions by binding to the intracellular MR, a ligand-activated transcription factor and member of the nuclear hormone receptor family, resulting in late genomic effects [36, 37] [10]. provided the first evidence for the notion that the MR agonist aldosterone stimulates the upregulation of substance P and somatostatin after adrenalectomy by a genomic mechanism. Moreover, aldosterone, through its interaction with the MR, increased transient receptor potential canonical 1 and 6 expression in the adrenal medulla of metabolic syndrome pigs [38–40]. In a similar way, aldosterone selectively increased Na^+^-K^+^-ATPase mRNA expression in rat hippocampus [11]. Similarly, chronic intra-cerebroventricular infusion of aldostrone synthase inhibitor FAD286 or MR blocker eplerenone reduced epithelial sodium channel C subunit expression in the supraoptic nucleus [12] and hypothalamus [13]. Taken together, this emerging evidence suggests that neuronal aldosterone may play a potential role in the transcriptional regulation of pain signaling molecules in peripheral sensory neurons.

A genomic effect of neuronal aldosterone is further supported by our demonstration of an enhanced translocation of MR from the cytosol to the nucleus of peripheral sensory DRG neurons. Following fractionation of homogenized DRG into a nuclear and a cytosolic compartment, MR were identified in both DRG of control rats and DRG of rats with an inflamed hindpaw. However, the intensity of the protein band was 8-fold higher in the nuclear than the cytosolic compartment under inflammatory pain conditions indicating an enhancement of nuclear translocation and, thus, supporting a genomic mechanism. This notion was confirmed by immunohistochemistry of the respective DRG showing an increased overlap of MR-immunorecativity with the nuclear marker DAPI and was supported by similar demonstrations of MR nuclear translocation within sensory afferent neurons of the cochlea [41] and striatum [42].

The next question arises: what are the functional consequences of blocking the 18-hydroxycorticosterone conversion into aldosterone via long-lasting inhibition of aldosterone synthase in nociceptive neurons? Since aldosterone content of peripheral sensory DRG was significantly elevated following inflammation, we examined whether i.t. infusion of aldosterone synthase inhibitor FAD286 reduced the aldosterone content in DRG of inflamed rats to control levels. Indeed, chronic i.t. infusion of FAD286 over 4 days significantly reduced aldosterone content as well as the number of aldosterone-IR DRG neurons in FCA rats. Consequently, this FAD286-mediated aldosterone reduction during FCA hindpaw inflammation not only reversed the enhanced expression of the pain signaling molecules TRPV1, CGRP, Nav1.8, and trkA, but also attenuated nociceptive behavior. Our findings of a transcriptional regulation of pain signaling molecules and subsequent nociceptive behavior are consistent with older studies [27, 28] which showed a long-lasting analgesic effect of MR antagonists.

## Conclusion

In summary, our present study demonstrates that long-lasting blockade of endogenous aldosterone from activating MR on sensory DRG neurons during local inflammation not only prevents the enhanced transcriptional expression of pain signaling molecules such as TRPV1, CGRP, Nav1.8, and trkA within peripheral sensory neurons but also subsequently attenuates mechanical hypersensitivity. This genomic effect of endogenous aldosterone during inflammatory pain was supported by the demonstration of an enhanced nuclear translocation of MR in peripheral sensory DRG neurons. Together, these findings suggest that local production of aldosterone within peripheral sensory DRG contributes to ongoing mechanical hypersensitivity via continuous activation of neuronal MR, most likely through genomically regulated enhanced expression of pain signaling molecules.

## Supplementary information

**Additional file 1: Table S1**. Characterization of primary antibodies used

## Data Availability

The datasets used and/or analyzed during the current study are available from the corresponding author (Shaaban.mousa@charite.de) on reasonable request. The authors will take responsible for maintaining availability.

## Abbreviations

MR: Mineralocorticoid receptors
FCA: Freund’s complete adjuvant
GR: Glucocorticoid receptors
PPT: paw pressure thresholds
DRG: Dorsal root ganglia
Can: Canrenoate-K
PARP: Poly (ADP-ribose) polymerase

## Footnotes

1 http://rsb.info.nih.gov/ij/. Last accessed November 28, 2018
