# Supplementary material for: Neuronal aldosterone elicits a distinct genomic response in pain signaling molecules contributing to inflammatory pain
Source: J Neuroinflammation. 2020 Jun 12;17:183. doi: 10.1186/s12974-020-01864-8 (PMC7291517; doi:10.1186/s12974-020-01864-8)
Supplement: Supplementary file 1 — Additional file 1: Table S1. Characterization of primary antibodies used [file 12974_2020_1864_MOESM1_ESM.doc]

**Table 1. Characterization of primary a**ntibodies used

| Antigen | Immunogen | Manufacturer, Species, Type, Catalogue Number | Dilution used |
| --- | --- | --- | --- |
| MR | generated against epitopes located at the N terminal of the MR molecule | Elise Gomez-Sanchez, Jackson,  USA, rMR 79–87 monoclonal antibody # Gomez-Sanchez et al., 2008 | 1.200 |
| Aldosterone | Aldosterone-3-CMO-BSA | (Novus Biologicals, LLC, CO, USA), rabbit polyclonal, # MB100-64658 | 1:2.000 |
| trkA | extracellular domain Ala33-Pro418 of rat trkA | R&D Systems (USA), goat polyclonal, # AF1056  # Shaqura et al., 2016 | 1:500 |
| TRPV1 | raised against C-terminus of the rat TRPV1 receptor (GSLKPEDAEVFKDSMVPGEK) | Neuromics (Edina, CA, USA), guinea pig polyclonal, Shaqura et al., 2014 | 1:1.000 |
| Na_V_1.8 | Fusion protein amino acids 1724-1956 (cytoplasmic C-terminus) of rat Nav1.8 | NeuroMab (University of California, Davis/NIH), mouse monoclonal, Cat # 75-166, RRID: AB_2183861 | 1:200 |
| CGRP | synthetic entire calcitonin gene-related peptide | Peninsula Laboratories (CA, USA), guinea pig polyclonal, # T-5027  # Mousa et al., 2013 | 1:1.000 |
